# Application and development of CRISPR technology in the secondary metabolic pathway of the active ingredients of phytopharmaceuticals

**DOI:** 10.3389/fpls.2024.1477894

**Published:** 2025-01-09

**Authors:** Haixin Gao, Xinyi Pei, Xianshui Song, Shiying Wang, Zisong Yang, Jianjun Zhu, Qiupeng Lin, Qinlong Zhu, Xiangna Yang

**Affiliations:** ^1^ College of Life Sciences, Northwest Agricultural and Forestry University, Yangling, Shaanxi, China; ^2^ Zhejiang Key Agricultural Enterprise Institute of Tiefengtang Dendrobium Officinale, Wenzhou, Zhejiang, China; ^3^ College of Resources and Environment, ABA Teachers College, Wenchuan, Sichuan, China; ^4^ College of Landscape and Hydraulic Engineering, Wenzhou Vocational College of Science and Technology, Wenzhou, Zhejiang, China; ^5^ College of Agriculture, South China Agricultural University, Guangzhou, Guangdong, China

**Keywords:** CRISPR, biosynthesis of active ingredients, gene editing, medicinal plants, secondary metabolism

## Abstract

As an efficient gene editing tool, the CRISPR/Cas9 system has been widely employed to investigate and regulate the biosynthetic pathways of active ingredients in medicinal plants. CRISPR technology holds significant potential for enhancing both the yield and quality of active ingredients in medicinal plants. By precisely regulating the expression of key enzymes and transcription factors, CRISPR technology not only deepens our understanding of secondary metabolic pathways in medicinal plants but also opens new avenues for drug development and the modernization of traditional Chinese medicine. This article introduces the principles of CRISPR technology and its efficacy in gene editing, followed by a detailed discussion of its applications in the secondary metabolism of medicinal plants. This includes an examination of the composition of active ingredients and the implementation of CRISPR strategies within metabolic pathways, as well as the influence of Cas9 protein variants and advanced CRISPR systems in the field. In addition, this article examines the long-term impact of CRISPR technology on the progress of medicinal plant research and development. It also raises existing issues in research, including off-target effects, complexity of genome structure, low transformation efficiency, and insufficient understanding of metabolic pathways. At the same time, this article puts forward some insights in order to provide new ideas for the subsequent application of CRISPR in medicinal plants. In summary, CRISPR technology presents broad application prospects in the study of secondary metabolism in medicinal plants and is poised to facilitate further advancements in biomedicine and agricultural science. As technological advancements continue and challenges are progressively addressed, CRISPR technology is expected to play an increasingly vital role in the research of active ingredients in medicinal plants.

## 1 Introduction

CRISPR/Cas9 technology, specifically the RNA-guided CRISPR/Cas9 nuclease system, represents a natural immune response found in bacteria and archaea that enables these organisms to resist the invasion of foreign viruses or plasmids. The immunity mechanism of this system relies on the complementary pairing of short repetitive sequences with invading DNA. By utilizing the cutting capability of the Cas9 protein, precise cleavage of foreign DNA is accomplished, thereby activating the cell’s DNA repair mechanisms (Guo et al., 2015). Among these repair pathways, non-homologous end Joining (NHEJ) is a method that may result in DNA sequence mismatch and gene inactivation, whereas homologous recombination repair (HDR) is a more precise repair mechanism that allows scientists to knock in or replace genes by introducing specific foreign DNA templates (Jiang et al., 2013). As CRISPR technology continues to evolve, researchers have developed two types of base editors: Cytosine base editor (CBE), which can convert cytosine into uracil, and adenine base editor (ABE), which can convert transform adenine into guanine (Rees and Liu, 2018). The discovery of these base editors has further broadened the application scope of CRISPR technology. Due to its straightforward principle, high editing efficiency, and theoretical applicability to various species, CRISPR technology has emerged as a vital tool in the field of gene editing. This technology not only offers scientists innovative methods for editing plant genes and studying active medicinal ingredients but also introduces convenience and new possibilities to related research fields, thereby advancing the progress of biomedicine and agricultural science (Ma et al., 2015).

Traditional Chinese medicinal plants constitute a significant component of human medical treatment and health care. With a long history, they have been utilized to address various diseases since ancient times. The statistical evaluation indicates that approximately 80% of the global population relies on traditional medicine to some degree, particularly in developing countries, where the use of medicinal plants is prevalent (Sofowora et al., 2013; Mbuni et al., 2020). Over 1,300 medicinal plants have been documented in Europe, with 90% sourced from wild habitats (Ssenku et al., 2022; Chen et al., 2016). Research underscores that many active ingredients in modern pharmaceuticals are derived from traditional Chinese medicinal plants. In the United States, about 9% of approved drugs are directly derived from plants, and this percentage is even higher on a global scale (Chen et al., 2016; Davis and Choisy, 2024). Consequently, Chinese medicinal plants serve not only as the foundation of traditional medicine but also as a vital resource for contemporary drug research and development. Secondary metabolites are diverse compounds synthesized during plant growth. Although they are not directly involved in plant growth and development, these compounds play a crucial role in the interactions between plants and their environment (Demain and Fang, 2000; Craney et al., 2013). They constitute a natural mechanism of plant defense against biotic and abiotic stresses by producing toxic or special-tasting substances, such as alkaloids, flavonoids, and terpenes, to defend against herbivores and pathogens (Zeeshan et al., 2022). Furthermore, many plant secondary metabolites, including flavonoids and polyphenols, not only serve functions within plants but are also believed to reduce the risk of chronic diseases, such as cardiovascular disease and certain cancers, due to their powerful antioxidant properties (Rao et al., 2023; Zeeshan et al., 2022). These compounds positively impact human health by enhancing metabolism, boosting immunity, and improving cellular function. Additionally, they contribute to ecosystem stability by attracting pollinators and beneficial organisms, thereby aiding in the reproduction and dispersal of plants (Guerriero et al., 2018; Pagare et al., 2015). Plant secondary metabolites are also of significant industrial importance, widely used in the production of fragrances, essences, and dyes, and are favored for their biocompatibility and low toxicity (Li et al., 2024). As the demand for natural products increases, research and development of these compounds have become a key area in drug development. Scientists continue to explore the potential for developing new drugs and treatments by investigating their synthesis pathways and biological functions (Thirumurugan et al., 2018). Advances in genomics and biotechnology have enabled more effective plant genetic improvement, focusing on the synthesis pathways of secondary metabolites, thus enhancing the plant’s ability to produce specific secondary metabolites through genetic engineering. This not only improves the plant’s disease resistance and nutritional value but also opens new avenues for the development of novel drugs (Pagare et al., 2015; Thirumurugan et al., 2018).

The diversity of plant secondary metabolites is extensive, encompassing a wide array of compounds such as alkaloids, terpenes, flavonoids, and polyphenols (Yang et al., 2016). These compounds have significant applications in medicine, agriculture, and industry. For instance, phenolic compounds can be categorized as non-polymeric polyphenols, oligomeric tannins, or polymeric tannins, depending on their degree of polymerization (Xiao et al., 2022). The principal active constituents of phenolic compounds include carnosolic acid, anthocyanins, rutin, lignans, flavonoids, and chlorogenic acid. These compounds are synthesized via a common precursor pathway known as the phenylacrylic acid metabolic pathway, hence they are referred to as phenylpropanoids in studies of plant secondary metabolism (Liu et al., 2020). Terpenoids, another class of secondary metabolites, are hydrocarbons composed of isoprene monomers. Their classification is based on the number of monomers, which leads to categories such as monoterpenes, sesquiterpenes, diterpenes, triterpenes, tetraterpenes, and polyterpenes. Each category exhibits unique biological activities and applications (Zhu et al., 2007). Monoterpenes and sesquiterpenes primarily constitute volatile oils, while diterpenes are the main components of resins. Triterpenes are crucial for the synthesis of plant saponins and resins, and tetraterpenes are predominantly fat-soluble pigments found widely in plants (Liang et al., 2017). Terpenoids are recognized for their anti-inflammatory and anti-tumor properties, highlighting their significance as active plant substances worthy of research. For example, artemisinin is noted for its antimalarial effects, and andrographolide is known for its antibacterial properties (Shi et al., 2018). Currently, research utilizing CRISPR technology is predominantly focused on terpenoids such as tanshinone, artemisinin, and ginsenosides. Alkaloids represent another class of nitrogen-containing organic compounds extensively found in medicinal plants. They are recognized for a variety of pharmacological effects, including antibacterial, anti-inflammatory, and antitumor activities, with some alkaloids also exhibiting analgesic properties. This class of compounds is widely utilized in clinical practice as significant active ingredients in medicinal plants (Feng et al., 2016). Well-known alkaloids include morphine, recognized for its analgesic properties, along with other alkaloids that exhibit various effects, such as cocaine, which acts as a stimulant, and ephedrine, known for its bronchodilator and stimulant effects (Wu et al., 2024; Kang and Wang, 2011). Pyridine alkaloids, like nicotine, primarily found in tobacco, are neurotoxic. Piperidine alkaloids, such as piperine in pepper, possess stimulating and anesthetic properties. It is important to note that quinoline is a heterocyclic compound that can be incorporated into the alkaloid structure but is not classified as an alkaloid itself (Hao et al., 2014; Sansone et al., 2023). Sulfur-containing compounds represent a small class of plant secondary metabolites, primarily consisting of certain biologically active compounds. Glucosinolates, predominantly found in cruciferous plants, exhibit anti-cancer and antibacterial properties; thiols, such as glutathione, play a role in the antioxidant response of plants and protect plant cells from oxidative stress damage (Kytidou et al., 2020). Polyketides are secondary metabolites generated through the polyketide synthesis pathway and exhibit a diverse range of biological activities. They are crucial as antibiotics and antifungals in plants, effectively shielding them from pathogens (Chen et al., 2024). Additionally, pigments such as anthocyanins contribute to the coloration of plants and play a vital role in photosynthesis (Teoh, 2015).

In recent years, there has been a growing discourse regarding the integration of traditional Chinese medicine with modern technology, which has sparked significant interest in employing gene editing techniques to investigate the medicinally active compounds found in plants. These compounds have emerged as a critical focus of scientific inquiry. Historically, research has primarily concentrated on elucidating the physiological and pharmacological functions of these medicinal active ingredients. However, with the maturation of genome sequencing technologies, researchers now possess a more comprehensive understanding of the genomes associated with plant secondary metabolism. This advancement has redirected research efforts towards the exploration of the functional genome or transcriptome within plant secondary metabolic pathways, aiming to investigate the synthesis of active substances at a more intricate molecular level (Chen et al., 2013). CRISPR technology, recognized as a powerful gene editing tool, has significantly contributed to the study of these active compounds. Compared to other technologies, CRISPR provides broader opportunities for genome exploration. The development of high-yielding medicinal and economically valuable plants can yield tangible societal benefits, such as enhanced efficacy and reduced costs. Despite its potential, the CRISPR/Cas9 system for genome editing is accompanied by a series of limitations and challenges. These issues include off-target effects, complex genome structures, low transformation efficiency, and insufficient understanding of metabolic pathways (Guo M. et al., 2022). To effectively harness CRISPR/Cas9 technology for plant genome editing, it is essential to address these challenges in order to fully realize the advantages of CRISPR technology. This article reviews the application of CRISPR technology in the study of plant active substances, discussing its potential, the problems that must be resolved, and future development directions. By overcoming these challenges, we anticipate that CRISPR technology will play a more significant role in future research on plant active ingredients, thus fostering the development and innovation of traditional Chinese medicine.

## 2 Common used CRISPR technology strategies in regulation of secondary metabolism

The secondary metabolism network of active substances in medicinal plants frequently involves interactions among various pathways, leading to the production of medicinal active substances being influenced by the expression levels of multiple genes across these pathways. To analyze the impact of gene networks on secondary metabolites and to enhance yields, it is essential to fine-tune the activities of relevant intermediate enzymes, ensuring that each reaction aligns with the overall biosynthetic pathway. A review of the latest research on CRISPR technology in the secondary metabolism of medicinal plants indicates that commonly employed strategies can be broadly categorized into enzyme regulation and transcriptional regulation. Furthermore, an extensive review of the literature reveals that enzyme regulation strategies are currently more prevalent in the secondary metabolism of medicinal plants compared to other approaches, such as transcriptional regulation. Additionally, most studies leverage CRISPR technology for gene knockdown to modulate the production of these metabolites (Naik et al., 2023; Mishra et al., 2023).

The principles of CRISPR gene editing can be extended to various regulatory mechanisms, including post-translational modifications, protein-protein interactions, the specificity of cis-regulatory elements, subcellular localization, and metabolite translocation, all of which contribute to plant metabolic editing. Among these regulatory mechanisms, pathway enzymes and transcription factors play the most critical roles. For metabolic pathway enzymes, multiple levels of regulation can be applied to metabolic editing, such as activating or inhibiting pathways and controlling flux into different branches of the pathway. In the case of transcription factors, metabolic editing can target various levels of regulation, such as their activity state (active or inactive) and their mode of action (whether functioning as a repressor or an activator).

### 2.1 Enzyme regulation

CRISPR/Cas9 technology facilitates gene editing by constructing single guide RNA (sgRNA) that is both homologous and complementary to the target sequence, thereby achieving precise positioning and accurate cutting for gene knockout or knock-in. The CRISPR/Cas9 system has been applied to a variety of medicinal plants, including dicots, tobacco, dendrobium, soybean, and radish (Alagoz et al., 2016; Hong et al., 2021; Kui et al., 2016; Sugano et al., 2018; Wang et al., 2019). In the context of plant gene editing, CRISPR technology is primarily utilized to modify genes and regulate the expression of various enzymes involved in the synthesis pathways of medicinal active ingredients. Key enzyme regulation strategies include enzyme inhibition, branched chain blockade, and modular classification of secondary metabolic pathways to investigate the functions of essential enzymes. The enzyme inhibition strategy employs the CRISPR system to silence enzymes within the biosynthetic pathway, thereby altering the content of target secondary metabolites (Zhou et al., 2012). This approach is the most prevalent strategy for studying plant secondary metabolic pathways and has been utilized in numerous projects investigating functional genes in secondary metabolite production pathways, including those related to salvianolic acids, tanshinones, anthocyanins, triterpene saponins, and cocaine. Examples of these studies include modifications of CRISPR technology for multi-gene editing, such as the double knockout of *Salvia miltiorrhiza* genes *SmDML3* and *SmMET1* using CRISPR/Cas9 technology, which facilitates the rapid and efficient knockout of target genes or entire gene families (Xing et al., 2014). Furthermore, one study employed CRISPR multiplex knockdown technology to simultaneously knock out two *XylT* genes and four *FucT* genes (12 alleles in total) that regulate plant-specific glycan glycosylation in tobacco, resulting in a deficiency of glycoproteins (Mercx et al., 2017). In another study, researchers utilized the CRISPR system to target and knock out the *NtPDS* and *NtPDR6* genes in tobacco (Gao J. et al., 2015), demonstrating that the CRISPR/Cas9 system is an effective tool for inducing targeted mutations within the tobacco genome. Similarly, the CRISPR/Cas9 system was employed to knock out β (1,2)-xylosyltransferase (*OsXylT*) and α (1,3)-fucosyltransferase (*OsFucT*) genes in rice, highlighting the versatility of CRISPR technology in plant genome editing (Jung et al., 2021).

Branched chain blocking strategies focus on obstructing or disrupting competing pathways within the synthesis pathway of the desired active substance, thereby diverting precursor substances to the target synthesis pathway. This method has been applied in the study of artemisinin and triterpene saponin metabolic pathways, specifically targeting the *SQS* and *CYP716A53v2* genes, respectively. This strategy is relevant not only to plants but also to the investigation of microbial cell factories. Recent studies on the metabolism of pharmaceutical active ingredients have identified simple organisms, such as *Escherichia coli* and *Saccharomyces cerevisiae*, as the primary hosts for genetic engineering. These organisms have been modified through gene editing to alter their original metabolic pathways, enabling low-cost production of target active substances (Huang et al., 2014). Notably, this included the use of CRISPR to enhance MVA metabolic flux in *Saccharomyces cerevisiae* cells while blocking the sterol branch pathway, which resulted in a significant increase in the content of the precursor mevalonate in the mutant strain (Jakočiūnas et al., 2015). Additionally, the group is collaborating with Amyris to engineer bacteria for high artemisinic acid production, thereby boosting the production of this compound (Paddon et al., 2013; Ro et al., 2006). This technology has also been applied to modify *S. cerevisiae* cells to develop engineered yeast capable of simultaneously synthesizing oleanolic acid and protoalcohol (Dai et al., 2014). With the support of CRISPR technology, multiple sites can be modified concurrently, significantly reducing operational complexity and facilitating research related to the secondary metabolism of active substances.

### 2.2 Transcriptional regulation

The CRISPR system-mediated transcriptional regulation represents a promising strategy for manipulating plant metabolic pathways, serving as a complement to enzymatic regulation. This approach offers the potential to overcome challenges associated with multienzyme regulation in plant secondary metabolic pathways. By enabling CRISPR-mediated control of transcription factors, researchers gain a novel method for investigating the biosynthetic pathways of plant secondary metabolites. Transcriptional regulation primarily encompasses activation and repression mechanisms. In 2015, the C-terminus of the inactive Cas9 protein was fused with the *VP64* transcriptional activation domain, resulting in the creation of a CRISPR-mediated transcriptional activator. This innovation has been successfully implemented in *Arabidopsis*, rice, and tobacco, demonstrating its functional efficacy (Lowder et al., 2015). Additionally, the 9-dCas PCO-3X (SRDX) synthetic transcriptional repressor has been utilized to repress both non-coding and protein-coding genes in *Arabidopsis* (Tang et al., 2017). Notably, this strategy enabled targeted mutagenesis of the *SlAN2* gene, an R2R3-MYB transcription factor linked to anthocyanin biosynthesis in the tomato genome, thereby confirming the role of *SlAN2* (Zhi et al., 2020). Transcription factors can manipulate metabolism by binding to cis-elements in promoter regions, thereby inhibiting or activating the expression of gene-encoded enzymes. For instance, in jasmonate biosynthesis, the transcription factor *CrMYC2* directly binds to the cis-element in the promoter of the *ORCA3* gene, regulating genes associated with terpene alkaloid biosynthesis. The CRISPR-mediated modification of the *ORCA3* promoter cis-element has been proposed as a strategy to enhance terpene alkaloid biosynthesis (Zhang et al., 2011). Additionally, CRISPR-a can be utilized to simultaneously activate multiple transcripts of factors, further promoting the synthesis of secondary metabolites. This approach enables researchers to modulate complex metabolic pathways and increase the content of medicinal components in plants. Transcriptional regulation is influenced not only by transcription factors but also by signal transduction pathways. Plants typically respond to environmental stimuli through a series of signaling molecules. By employing CRISPR technology, researchers can interfere with these signaling pathways, thereby affecting the synthesis of secondary metabolites. For example, editing genes involved in a plant’s stress response can enhance the plant’s resilience while improving its ability to synthesize medicinal ingredients. Recent advancements in the transcriptional regulation of plant secondary metabolism have underscored the pivotal role of CRISPR gene editing technology in promoting research in this field and exploring pathways to human health.

### 2.3 Regulation of signaling pathways

Signaling pathways play a crucial role in plant growth, development, and secondary metabolism. By sensing environmental changes, plants regulate their internal signaling pathways, which in turn affects the synthesis of secondary metabolites. Under stress conditions such as drought, salinity, and alkalinity, plants activate specific signaling pathways to adapt to their environment. These pathways often involve the synthesis and signal transduction of hormones, including abscisic acid and cytokinins. For instance, CRISPR technology can be employed to edit genes associated with abscisic acid synthesis, thereby enhancing plant resistance to stress and promoting the synthesis of related secondary metabolites (Guo M. et al., 2022). The role of plant hormones in secondary metabolism is significant and should not be overlooked. Through CRISPR technology, scientists can disrupt hormone signal transduction pathways and regulate plant responses to hormones, thus influencing the accumulation of secondary metabolites. Manipulating the balance between auxin and cytokinin can affect plant growth patterns and the synthesis of active compounds (Al Aboud, 2024). The synthesis of secondary metabolites typically results from intricate interactions among multiple signaling pathways. The application of CRISPR technology enables researchers to unravel these complex interaction networks and identify key genes and signaling pathways. In certain medicinal plants, studies have demonstrated cross-regulation among different signaling pathways. By utilizing CRISPR technology, these critical nodes can be precisely edited to optimize the production of secondary metabolites (Guo M. et al., 2022; Kumar et al., 2023).

## 3 Application of CRISPR technology in secondary metabolism of medicinal plants

**Figure 1:**
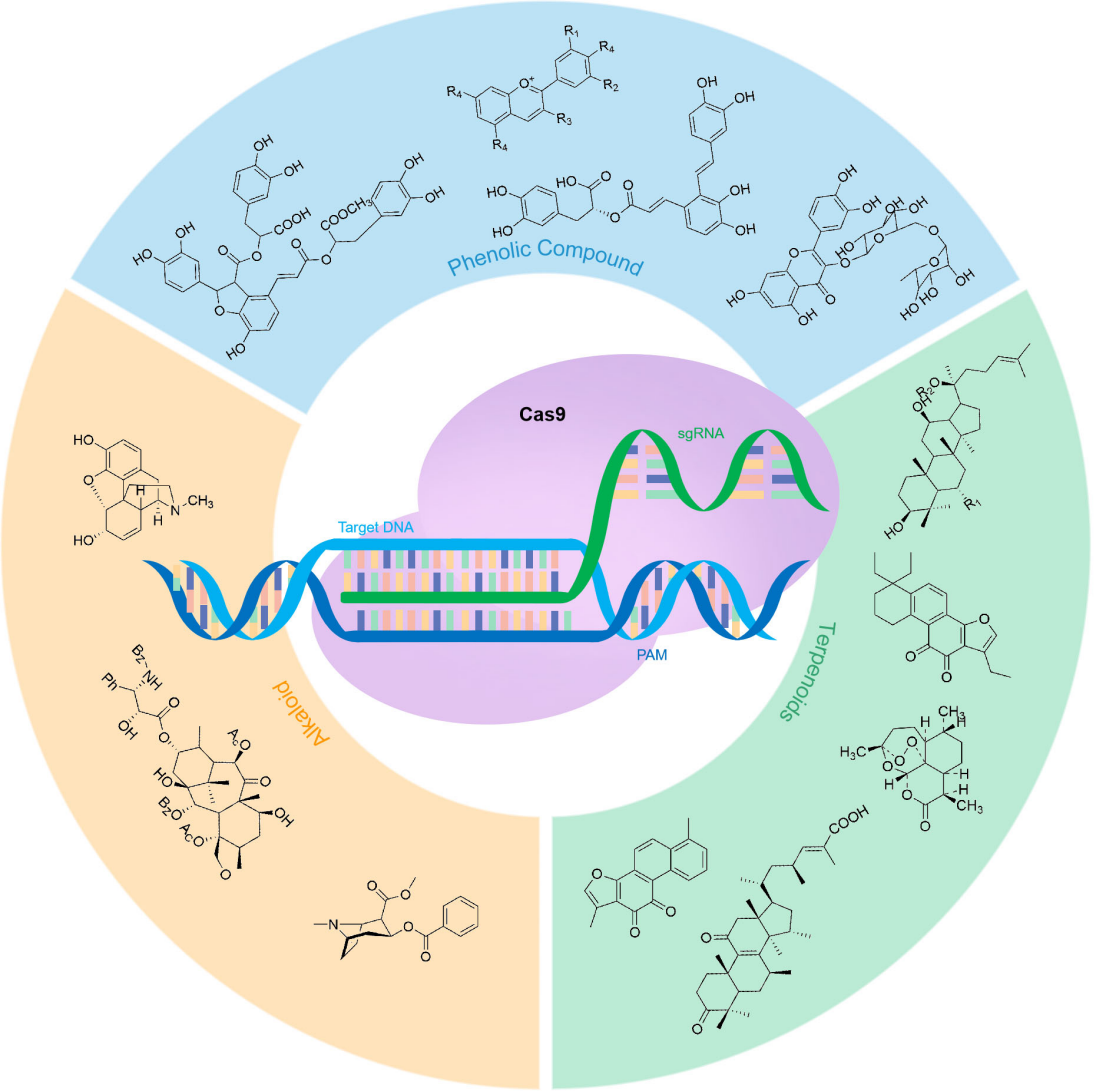
CRISPR/Cas9 system in secondary metabolically active substances.

Numerous landmark studies have utilized CRISPR technology to modify plant metabolism. The methods employed and their corresponding examples are discussed in detail below (**Figure 1**; **Table 1**).

**Table 1:** Application of CRISPR technology in medicinal plants.

Active substances		Species	Functioning genes	Strategies		Impact	References
Phenols	Salvianolic acid	*Salvia*	*SmRAS*	Enzyme	Enzyme	Decrease	Zhou et al. (2018)
		*miltiorrhiza*	*SmLACs*	regulation	inhibition	Decrease	Zhou et al. (2021)
			*SmPAL1*			Decrease	Qiu (2018)
			*SmLAC*			Decrease	Feng (2019)
			*SmCYP76AK5*			Decrease	Yu (2019)
			*SmbZIP1*			Decrease	Mu et al. (2015)
	Anthocyanins	*Vitis vinifera*	*VvbZIP36*			Increase	Tu et al. (2022)
		*Solanum lycopersicum*	*SlAN2*	Transcriptional regulation	Transcriptional repression	Decrease	Zhi et al. (2020)
	Rutin	*Fagopyrum esculentum*	*FtMYB45*	Enzyme	Enzyme	Increase	Wen et al. (2022)
Terpenoids	Tanshinone	*Salvia*	*SmCPS1*	regulation	inhibition	Decrease	Li et al. (2017)
		*miltiorrhiza*	*SmCYP76AK2*			Decrease	Li et al. (2021)
			*SmCYP76AK3*			Decrease	Li et al. (2021)
			*SmDML3 and SmMET1*		Enzyme	Decrease	Li et al. (2018)
			*SmbZIP1*		double	Increase	Mu et al. (2015)
			*SmWRKY34*		knock	Increase	Gao K. et al. (2015)
			*SmbZIP3*			Decrease	Gao K. et al. (2015)
			*SmJAZ9*			Increase	Wang et al. (2013)
			*SmMYB76*			Decrease	Wang et al. (2013)
			*SmbHLH60*			Increase	Chen et al. (2012)
	Artemisinin	*Artemisia annua L.*	*AaSQS*		Clogged branch lines	Increase	Koerniati and Simanjuntak (2020)
	Triterpene	*Panax ginseng C. A. Meyer*	*PgCYP716A53v2*		Enzyme	Increase	Yao et al. (2022)
	Saponins	*Trifolium repens L.*	*TfCYP93E2*		inhibition	Decrease	Confalonieri et al. (2021)
			*TfCYP72A61*			Decrease	Confalonieri et al. (2021)
Alkaloids	Cocaine and morphine	*Papaver somniferum L.*	*Ps4/OMT2*			Decrease	Alagoz et al. (2016)

### 3.1 Phenolic compounds

Salvianolic acid is a significant active component of *Salvia miltiorrhiza*, renowned for its medicinal properties, which includepromoting blood circulation, alleviating blood stasis, relieving pain, clearing heat, cooling the blood, and eliminating carbuncles. This compound holds considerable medicinal and economic value (Liang et al., 2016). The biosynthesis of phenolic acid in *Salvia miltiorrhiza* occurs via the phenylpropanoid pathway, with research primarily focusing on the functional enzymes involved in tanshinones and phenolic acid. The researchers employed CRISPR technology to target the *RAS* gene, which plays a crucial role in phenolic acid synthase activity, achieving a mutation rate of approximately 50%. This led to a corresponding decrease in both phenolic acid content and RAS expression levels (Zhou et al., 2018). Similar studies were conducted on the knockout of the *SmLACs* gene to investigate their role in phenolic acid metabolism, revealing that the growth of hairy roots was significantly inhibited and that lignin content was nearly undetectable, alongside a reduction in the accumulation of rosmarinic acid and salvianolic acid B (Zhou et al., 2021). Furthermore, the researchers utilized CRISPR technology to examine the *SmPAL1* and *SmLAC* gene families in the secondary metabolism of *Salvia miltiorrhiza* (Feng, 2019, 58-70; Qiu, 2018, 32-38). These findings illustrate that the CRISPR/Cas9 system can effectively identify important genes within plant gene families and serves as an efficient and specific genome editing tool for *Salvia miltiorrhiza*. As a medicinal plant with a well-characterized genome, *Salvia miltiorrhiza* possesses a mature genetic transformation system, which offers a promising avenue for future research on metabolism of active substance using CRISPR technology. Recently, an enhanced gene transformation system known as Cut-Dip-Budding (CDB) has been developed, effectively promoting the application of CRISPR/Cas9 in gene functional analysis and breeding (Cao et al., 2024). Additionally, a protoplast-plant regeneration system for *Salvia miltiorrhiza* has been successfully established, enabling gene-free CRISPR/Cas9-mediated gene editing that can target one or multiple sites (Hsu et al., 2024). The advancement of plant genome editing technology now benefits from a streamlined and efficient genetic transformation systems, which promise to accelerate the discovery and enhancement of functional genes, particularly for medicinal plants that have traditionally posed challenges for transformation. CRISPR technology has the potential to significantly lower production costs per unit of product by increasing throughput and reducing production time. Conventional methods for extracting salvia acid often necessitate substantial quantities of raw materials and involve complex extraction processes. In contrast, genetic engineering allows for the synthesis of the same compounds in microorganisms, thereby decreasing reliance on natural plant sources. An optimized biosynthetic pathway can yield higher quality and greater quantities of salvianic acid, thereby meeting market demand. These advancements have notably enhanced the medicinal value of *Salvia miltiorrhiza* and its application in the treatment of cardiovascular diseases.

Anthocyanins are common secondary metabolites found in plants, known for their antioxidant and free radical scavenging properties. They are widely utilized in the food, pharmaceutical and beauty industries (Vimolmangkang et al., 2013). Plants serve as the primary source for anthocyanin extraction, and numerous studies have investigated the application of CRISPR technology to regulate the anthocyanin content in these organisms. For instance, CRISPR technology was employed to knock out the *VvbZIP36* gene, identified as a negative regulator of anthocyanin accumulation within the grape bZIP family, thus promoting the accumulation of related metabolites (Tu et al., 2022). Additionally, CRISPR was utilized to induce targeted mutagenesis of the tomato gene *SlAN2*, an R2R3-MYB transcription factor associated with anthocyanin biosynthesis in the tomato genome. The study revealed that, compared to wild-type plants, the fruit weight and anthocyanin content of the mutant were reduced, thereby confirming the role of *SlAN2* in regulating anthocyanin accumulation (Zhi et al., 2020). Beyond grapes and tomatoes, the CRISPR/Cas9 system successfully edited three key genes in the anthocyanin biosynthetic pathway of black rice—*OsF3H*, *OsDFR* and *OsLDOX*—with an editing efficiency of 56.7%. This targeted modification led to significant changes in seed color and anthocyanin content in the mutant strains (Jung et al., 2019). These results demonstrate that the CRISPR/Cas9 system can effectively induce substantial gene-specific mutations in black rice, suggesting its potential application in the breeding of crops and medicinal plants in the future. CRISPRi technology-mediated silencing of the transcriptional repressor MetJ can relieve the inhibition of the methionine biosynthetic pathway, thereby increasing the availability of S-adenosylmethionine (SAM) and promoting the production of O-methylated anthocyanins, with output increasing to 51 mg/L. This result represents a two-fold improvement compared to the non-targeting CRISPRi control strain, and an overall enhancement of 21-fold (Cress et al., 2017). To detect the presence of the transgene, the researchers combined CRISPR/Cas9 gene-editing technology with a system that activates anthocyanin biosynthesis, providing a visible signature. This anthocyanin tag-assisted CRISPR (AAC) technology enables the identification of transgenic events at the callus stage, the selection of transformants with high Cas9 expression, and the identification of non-transgenic plants in the field. The researchers utilized AAC technology to edit the *LAZY1* and *G1* genes, successfully generating numerous transgenic-free and targeted gene-edited plants in the T1 generation, thereby significantly reducing the labor, time, and cost associated with editing target genes in rice (He et al., 2020). Anthocyanins are directly associated with plant coloration, prompting some studies to utilize the CRISPR/Cas9 system to investigate the underlying mechanisms responsible for the blue flowers observed in Japanese gentian plants, specifically the accumulation of polyacylated anthocyanins known as gentiodelphin. By knocking out three anthocyanin-modifying genes (*Gt5GT*, *Gt3′GT*, and *Gt5/3′AT*), the flower colors produced by the transgenic lines exhibit variations of light reddish-purple, light pink, and lavender, respectively, which contrast with the bright blue of wild-type plants. The differences in flower coloration are significant. The glycosylation and subsequent acylation of the 3′-hydroxyl group on the B ring are essential for the development of blue gentian flowers (Tasaki et al., 2019). Furthermore, CRISPR/Cas9-mediated multiple gene editing technology can also be employed to target the *PSY1*, *MYB12*, and *SGR1* genes associated with fruit color formation in tomatoes. This approach has successfully transformed the red-fruited tomato variety ‘Ailsa Craig’ into tomatoes exhibiting a range of fruit colors, including yellow, brown, pink, light yellow, pinkish brown, chartreuse, and light green. This strategy is more time-efficient than traditional breeding methods and yields GMO-free plants with varied fruit colors in less than a year (Yang et al., 2022a). Through precise gene editing, researchers can effectively enhance the anthocyanin content in plants and elevate fruit quality. As technology continues to advance, CRISPR technology is expected to play an increasingly significant role in future plant breeding, offering new solutions for the sustainable development of global agriculture.

Flavonoids represent a significant class of natural compounds that are abundantly present in various plants, particularly in fruits, vegetables, grains, bark, roots, stems, flowers, tea, and wine. These compounds exhibit diverse phenolic structures and frequently play crucial roles in plant growth, development, and resistance to environmental stressors. Furthermore, flavonoids are recognized for their potential health benefits in humans, exhibiting biological activities such as antioxidant, anti-inflammatory, and anti-cancer properties (Chen et al., 2023). Rutin, a natural flavonoid glycoside commonly referred to as vitamin P, possesses anti-allergic, antioxidant, anti-inflammatory, and antiviral properties (Yang et al., 2022b). As a valuable component of traditional Chinese herbal medicine, buckwheat is particularly rich in rutin and serves as its primary source. By employing CRISPR/Cas9-mediated targeted mutagenesis of the *FtMYB45* gene, researchers achieved a mutation rate of 50%, resulting in increased levels of rutin, catechins, and other flavonoids in the trichome mutants (Wen et al., 2022). This innovative technology paves the way for advancements in functional gene research and the genetic enhancement of buckwheat. The *GmF3H1*, *GmF3H2*, and *GmFNSII-1* genes in soybeans, which are involved in the flavonoid biosynthetic pathway, successfully increased the content of soybean isoflavones through CRISPR/Cas9-mediated multiple gene editing and enhanced resistance to soybean mosaic virus (SMV) (Zhang et al., 2020). Concurrently, the key flavonoid compound UDP-2-O-glucose transferase (*MdPGT1*) in apples can be knocked out using CRISPR/Cas9 gene editing technology, which reduces the accumulation of the flavonoid phloridzin in apple leaves without adversely affecting plant growth. This outcome sharply contrasts with the growth inhibition and leaf morphological changes induced by traditional transgenic methods, such as RNA interference (RNAi), when *MdPGT1* is downregulated. Furthermore, the differential regulation of phytohormones may be associated with the effects of phloridzin reduction on growth (Miranda et al., 2023). The flavonoid biosynthetic pathway genes in rapeseed (*Brassica napus L.*) have also been studied using CRISPR/Cas9 technology to investigate their roles in seed color, oil content, and fatty acid composition. Mutations in these genes lead to alterations in seed color, an increase in seed oil content, and a reduction in the accumulation of pigments and lignin in the seed coat, which may facilitate further rapeseed breeding (Li et al., 2024). Additionally, CRISPR interference (CRISPRi) technology has been applied to downregulate the expression of the cinnamic acid-4-hydroxylase (C4H) gene in tobacco, where *C4H* serves as a key enzyme in the flavonoid biosynthetic pathway. Silencing the *C4H* gene using CRISPRi technology can enhance flavonoid biosynthesis in tobacco (Karlson et al., 2022). Additionally, multiple knockout mutant lines of the tomato *HQT* gene were generated through the CRISPR/Cas9 system. These slhqt (Solanum lycopersicum *HQT* knockout) mutants do not accumulate CGA or other caffeoylquinic acids (CQAs) in various parts of the plant, including fruits, stems, leaves, flowers, and roots. This finding suggests that CQA biosynthesis in tomato is predominantly reliant on the *HQT* pathway, a dependence that may also extend to other Solanaceae crops. Furthermore, the study revealed that the absence of CGA in slhqt mutant plants resulted in elevated levels of hydroxycinnamoyl-glucose and flavonoids compared to control plants (D'Orso et al., 2023). These results provide valuable insights into the role of *HQT* in plants and may have significant implications for crop improvement and stress response research.

### 3.2 Terpenoids

Tanshinone is the primary lipophilic bioactive component of the Chinese herbal medicine derived from the rhizome of *Salvia miltiorrhiza*, serving as a significant intersection between traditional Chinese medicine and advanced molecular biology. Research has demonstrated that Tanshinone exhibits a range of biological activities, including antibiotic properties, as well as anti-inflammatory and antioxidant effects (Gao et al., 2009). Preparations of sage are currently undergoing phase II clinical trials for the treatment of cardiovascular disease (Chen et al., 2014). Since its initial application in 2017, CRISPR technology has been employed to knock out the *SmCPS1* gene in*Salvia miltiorrhiza*, facilitating the investigation of the gene’s role in the tanshinone and the changes in tanshinone production pathway and alterations in tanshinone content. This represents the first instance of CRISPR technology being utilized to study *Salvia miltiorrhiza*, heralding a new era in the exploration of metabolic processes (Li et al., 2017). Additionally, this technology has been applied to knock out the enzyme-related genes *SmCYP76AK2* and *SmCYP76AK3* in *Salvia miltiorrhiza*, with the objective of examining their influence on tanshinone synthesis (Li et al., 2021). Subsequently, CRISPR technology successfully knocked out the *SmCYP76AK5* gene, which is involved in the tanshinone biosynthesis pathway, revealing a reduction in tanshinone levels in mutant plants (Yu, 2019, 34-40). Studies have demonstrated that the *SmMYB98* transcription factor is highly expressed in the lateral roots of *Salvia miltiorrhiza* and can be either knocked out (KO) or overexpressed (OE) using CRISPR/Cas9 technology. Experimental results indicate that the overexpression of *SmMYB98* leads to an increase in the accumulation of Tanshinones and Salvianolic acids, whereas knockout of this factor results in a reduction of these metabolites. Furthermore, *SmMYB98* also plays a negative regulatory role in gibberellin biosynthesis (Hao et al., 2020). Single gene editing in plants utilizing CRISPR technology is primarily focused on secondary metabolic systems. Notably, the same active substance can be associated with different metabolic pathways, which often involve complex regulatory relationships among enzymes. Moreover, a single enzyme may be regulated by multiple genes. CRISPR stands out as a highly efficient and rapid gene editing technology, demonstrating significant potential in the realm of multigene editing. The principle underlying CRISPR multiple gene editing involves constructing multiple sgRNAs within a single vector for transformation and expression, achieved through techniques such asGolden Gate cloning or Gibson assembly. These sgRNAs are then combined with the Cas9 protein to simultaneously target and knock down multiple genes using CRISPR technology (Ma et al., 2016). Researchers have reported employing the Golden Gate assembly strategy to construct sgRNAs targeting the Salvia genes *SmDML3* and *SmMET1* within the same vector. This approach utilizes CRISPR/Cas9 technology to achieve a double knockdown of these genes, demonstrating higher mutation efficiency compared to single-gene editing with CRISPR (Li, 2018, 122-129). The metabolome and transcriptome were analyzed by comparing two red root systems and one white root system of *Salvia miltiorrhiza*. The study found that the content of 18 Tanshinones in the white roots was significantly lower than that in the other roots, and the expression of 5 genes related to Tanshinone biosynthesis was significantly down-regulated in white roots, thereby affecting the metabolic flow of Tanshinone. The research speculates that this may explain why exhibits white roots. These findings not only elucidate the formation mechanism of root color in *Salvia*, but also provide an effective approach for exploring the biosynthetic pathway of Tanshinone. This study revealed the potential mechanisms underlying Tanshinone biosynthesis in *Salvia miltiorrhiza* by integrating metabolome and transcriptome analyses, and employed CRISPR/Cas9 technology to investigate and validate the functions of related genes, thereby establishing a foundation for genetic improvement and synthetic biology strategies in *Salvia miltiorrhiza* (Su et al., 2021). CRISPR technology holds significant potential for enhancing the secondary metabolism pathways of tanshinone. By employing gene editing, researchers can reconstruct metabolic pathways and explore regulatory mechanisms, thereby optimizing the synthesis of tanshinone and increasing both its yield and medicinal value. Furthermore, the ongoing advancement of this technology is expected to unlock even greater opportunities for the future of plant metabolic engineering, facilitating more efficient biosynthesis and resource utilization.

Malaria is a globally prevalent insect-borne disease, with the highest incidence occurring in Africa (Hu et al., 2021). Artemisinin, an active component derived from the plant Artemisia annua, is effective in treating malaria and has also been reported to possess antiviral, anticancer, and anti-schistosomiasis properties. This compound has been the subject of extensive research, including investigations utilizing CRISPR technology (Abdin et al., 2003). Specifically, CRISPR/Cas9 has been employed to enhance artemisinin content by disrupting the sterol synthesis pathway (Koerniati and Simanjuntak, 2020). Both artemisinin and sterols are synthesized from the common precursor farnesyl diphosphate (FDP). The gene squalene synthase (SQS), which regulates sterol biosynthesis, is considered a competing gene in artemisinin biosynthesis. The strategy of promoting artemisinin production by employing CRISPR to knock out the *SQS* gene. Currently, the primary sources for artemisinin extraction are the herb Artemisia annua and microbial cultures. The artemisinin biosynthetic pathway in Bacillus subtilis is compartmentalized into three distinct modules: a terpene synthase module, a branching pathway module, and a central metabolic module. The researchers utilized CRISPR technology to enhance genes related to artemisinin synthesis, targeting the TCA cycle, MEP pathway, branched pathway, and terpene synthase pathway. This strategy offers a novel approach to increasing terpenoid content in Bacillus subtilis (Song et al., 2021). Researchers are investigating transcription factors that play a crucial regulatory role in the biosynthesis of artemisinin and flavonoids. They employ CRISPR/Cas9 technology to edit the R2R3-MYB transcription factor encoded by the *AaTAR2* gene. Knocking out *AaTAR2* significantly reduces artemisinin content, while its overexpression leads to a notable increase in artemisinin levels. This indicates that *AaTAR2* serves a positive regulatory function in artemisinin biosynthesis and may influence artemisinin production by affecting the development of glandular secretory hairs (Zhou et al., 2020). As noted by Ikram and Simonsen, scientists are exploring various bioengineering techniques to enhance the yield of Artemisia annua. These techniques encompass genetic modification of *A. annua* itself and heterologous expression in other plant systems, such as tobacco and mosses (Ikram and Simonsen, 2017). The primary focus of these efforts has been on enhancing the expression of key enzymes in the artemisinin biosynthetic pathway and inhibiting competing pathways through metabolic engineering, thereby redirecting more metabolic flow toward artemisinin synthesis. These advancements not only offer new strategies for artemisinin production but also provide valuable insights and methodologies for the production of other plant-derived natural products.

The triterpenoid ganoderic acids (GAs) derived from Ganoderma lucidum represent a valuable component of traditional Chinese herbal medicine, known for their anti-tumor properties. Additionally, GAs serve as critical indicators of the quality of Ganoderma lucidum and its associated products. Researchers have successfully developed a CRISPR/Cas9 editing system aimed at functional genes involved in GA biosynthesis, resulting in a significantly increase in the GA content (Wang et al., 2020). This technology is also employed to knock out key genes within the GA biosynthesis pathway, including *CYP5150L8*, as well as the homologous genes *glcrz1* and *glcrz2*, which are associated with the calcium signal transduction factor CRZ1. The objective is to elucidate their roles in the influence of calcium signaling on GA biosynthesis. The study revealed that, in comparison to the wild-type strain, the *glcrz1*-knockout strain did not exhibit increased GA production following the addition of Ca^2+^. Conversely, the *glcrz2*-knockout strain demonstrated severely impaired hyphal growth and GA synthesis upon Ca^2+^ supplementation. (Zhang Y. et al., 2023; Li and Zhong, 2020) These findings hold significant implications for molecular breeding and biotechnological applications of Ganoderma lucidum, offering substantial potential to inform future research and developments in this area.

Triterpene saponins, a class of steroidal compounds recognized for their anticancer effects, are commonly referred to as ginsenosides. However, their natural abundance in ginseng is significantly limited. Consequently, enhancing the accumulation of triterpene saponins through gene editing techniques has become a major focus of research on secondary metabolites in ginseng (Sparg et al., 2004). In 2022, researchers reported the groundbreaking application of the CRISPR/Cas9 system to induce targeted mutations in the ginsenoside biosynthetic pathway (Choi et al., 2022). They constructed a PPT-type ginsenoside-deficient mutant, which produced only PPD-type saponin through CRISPR/cas9-mediated mutagenesis of the ginseng *PPT* synthase gene. *PPT* synthase in ginseng catalyzes the conversion of *PPD saponins in*to *PPT* saponins, and significant differences exist in the pharmacological activities between these two types of saponins. Therefore, novel pure PPD ginseng mutants may offer new medicinal value compared to wild-type ginseng. In the same year, a CRISPR-mediated regulatory strategy for the ginsenosides biosynthesis pathway was also reported. This study combined the overexpression of ginsenoside synthesis genes squalene cyclooxygenase, *Pq3-O-UGT2* and *PAL* with CRISPR/cas9 knockdown of *CYP716A53v2* to block branched shunting in the ginsenoside production pathway, thereby enhancing the accumulation of ginsenoside Rg3 (Yao et al., 2022). Triterpene saponins are not only abundant in ginseng but also play a significant role in the active substances of alfalfa plants.CRISPR/cas9 technology was employed to knock out *CYP93E2* and *CYP72A61* genes associated with soybean saponinol B biosynthesis in alfalfa plants, achieving an editing efficiency of 84% (Confalonieri et al., 2021). The results indicate that all *CYP93E2* knockout mutants do not produce saponin elements, instead shifting metabolic flux to generate valuable saponin compounds. Furthermore, studies utilizing CRISPR-Cas9 technology have targeted the glycosyltransferase gene (*GuCSyGT*) derived from cellulose synthase in licorice. The complete deletion of soybean saponin I confirms the role of *GuCSyGT* in the biosynthesis of triterpene saponins in plants, providing new insights for studying the physiological functions of plant saponins (Sakanishi et al., 2023).

### 3.3 Alkaloids

Paclitaxel, a complex diterpene alkaloid with inherent anticancer properties, has emerged as a prominent active compound in medical research in recent years (Weaver, 2014). CRISPR technology has significantly contributed to the investigation of paclitaxel biosynthesis and drug resistance. Specifically, researchers utilized CRISPR-guided DNA methylation technology to knock down phenylalanine ammonia lyase (PAL), which is the initial enzyme in the phenylpropanoid pathway that regulates paclitaxel biosynthesis in yew, resulting in a notable increase in paclitaxel accumulation (Brzycki Newton et al., 2023). Additionally, in triple-negative breast cancer (TNBC) cell models, scientists identified multiple gene targets associated with paclitaxel resistance, including *ATP8B3*, *FOXR2*, *FRG2*, and *HIST1H4A*, through a combination of *in vitro* and *in vivo* genome-wide CRISPR screening methods. The deletion of these genes was found to enhance TNBC cell resistance to paclitaxel and has been recognized as a negative regulator of cancer stem cell properties (Yan et al., 2023). Furthermore, researchers employed the CRISPR/Cas9 system to integrate various *GGPP* synthase genes into the yeast genome, establishing a biotechnological platform for the synthesis of paclitaxel biosynthetic precursors. This engineered strain may facilitate the identification of candidate genes for future microbial paclitaxel biosynthesis (Utomo et al., 2021). Collectively, these studies underscore the substantial potential of CRISPR technology to augment paclitaxel production and address challenges related to cancer drug resistance.

Cocaine and morphine are primarily derived from the opium poppy, which is recognized in traditional Chinese medicine for its properties that strengthen the spleen, enhance appetite, clear heat, and detoxify. It is commonly employed in the treatment of diarrhea, dysentery, and reflux (Kang and Wang, 2011). In 2016, CRISPR technology was first applied to the opium poppy, utilizing viral TRV to successfully knock out the *4*′*OMT2* gene and deliver the CRISPR/Cas9 complex into target cells. This innovative method was employed to investigate the function of the *4*′*OMT2* gene within the biosynthetic pathway of pharmaceutically active substances in the opium poppy, leading to a significant reduction in the production of cocaine and morphine in the resulting mutant plants (Alagoz et al., 2016). Nicotine is a compound characterized by a unique structure and a range of biological activities. It exerts diverse effects on the nervous system, influencing addiction, anxiety, cognitive function, and various other aspects. While it holds potential for certain medical applications, nicotine is also associated with significant health risks. Research on nicotine not only enhances our understanding of its biological effects but also yields crucial insights for public health policy and smoking cessation treatments (Sansone et al., 2023). Specifically, CRISPR/Cas9-induced mutations in the *A622* and *BBL* genes have a marked effect on nicotine biosynthesis in tobacco. These mutations not only reduce the levels of nicotine and other alkaloids but also result in developmental abnormalities in the plants (Jeong et al., 2024). Additionally, the establishment and application of the CRISPR/Cas9 system in wild tobacco (*Nicotiana alata*) have led to significant advancements in genetic transformation methods. By optimizing these methods, we achieved a notable increase in genetic transformation efficiency and successfully performed genome editing in *N. alata* (Yuan et al., 2024). Furthermore, an optimized CRISPR/Cas9 system was employed to enhance gene editing efficiency in tobacco. By utilizing the OsU3-tRNA promoter combination to drive sgRNA expression and incorporating a visible Ros1 expression cassette for monitoring transgenic events, we simplified the screening process and improved overall efficiency (Zhang J. et al., 2023).

### 3.4 Glycosides

Glycosides are a class of compounds commonly found in plants, comprising sugar molecules and non-sugar moieties, referred to as aglycones, which are linked by glycosidic bonds. These compounds can be categorized into various groups based on the type of sugar and the characteristics of the aglycone. Due to their diverse biological activities, glycosides play a significant role in drug development and therapeutic applications (Kytidou et al., 2020). Transgenic technology and CRISPR-Cas9 gene editing were employed to knock out the *MYB28* gene in broccoli, resulting in a reduction of glucoside accumulation (Zhang et al., 2024). This demonstrates that CRISPR-Cas9 technology can effectively induce mutations in specific genes within the broccoli genome, which is significant for further validating gene functions and achieving precise trait improvement. Additionally, the *BoaAOP2* gene in Chinese kale was edited using the CRISPR/Cas9 system. This gene encodes a 2-oxoglutarate-dependent dioxygenase that catalyzes the conversion of glucoside (GRA) into gluconaphthyridine (GNA). In the edited T1 generation mutant, the GRA content increased significantly, while the GNA content and total aliphatic glucosinolate (GSL) levels decreased. These findings suggest that targeted editing of the *BoaAOP2* gene via CRISPR/Cas9 alters the metabolic flux of aliphatic GSL side chains and enhances GRA content in Chinese kale (Zheng et al., 2023). A high-efficiency broccoli genetic transformation system mediated by PEG-Ca^2+^ was utilized to investigate the subcellular localization of the *FMOGS-OX5* gene, which is associated with glucoside synthesis, as well as the clubroot resistance gene *CRa*. Both genes are expressed in the cytoplasm and nucleus, providing a scientific basis for studying the regulation of glucoside metabolism and clubroot disease resistance in cruciferous crops (Zhao et al., 2023). Utilizing the CRISPR/Cas9 system, the phytoene desaturase (BoPDS) gene in cabbage, which is critical to the carotenoid biosynthetic pathway, was edited. The observation of albino seedlings in the T0 generation suggests that the *BoPDS* gene may have been successfully edited. Additionally, mutations in the *Bol016089* gene, which shares high homology with the *BoPDS* gene, were detected, indicating that the CRISPR/Cas9 system can edit multiple genes simultaneously (Ma et al., 2019). By employing the CRISPR/Cas9 system alongside the endogenous tRNA processing system, multiple genes in cabbage—including *BoPDS*, *BoSRK*, and *BoMS1*—were edited to achieve multi-site and multi-gene mutations. Notably, mutations in the *BoSRK3* gene completely suppressed self-incompatibility, transforming self-incompatible lines into self-compatible lines. Furthermore, mutations in the *BoMS1* gene resulted in completely male-sterile mutants, which can be economically propagated through bee-mediated cross-pollination (Ma et al., 2019).

### 3.5 Polyketides

Polyketides represent a substantial class of structurally diverse secondary metabolites that are prevalent in microorganisms, plants, and animals. These compounds play significant ecological roles within organisms and are extensively utilized in medicine, agriculture, industry, and other domains owing to their varied biological activities. The CRISPR interference (CRISPRi) system is employed to finely regulate central metabolic pathways in *Escherichia coli*, thereby enhancing polyketide biosynthesis. By silencing candidate genes using synthetic sgRNA, intracellular levels of malonyl-CoA were successfully increased, leading to an augmentation in the production of the plant-specific secondary metabolite (2S)-naringenin (Wu et al., 2015). The CRISPR-Cas9 system facilitates metabolic engineering by enabling the deletion and insertion of biosynthetic gene clusters (*BGCs*) to enhance polyketide production. Additionally, the CRISPR-BEST (CRISPR-Base Editing System) has been developed for efficient base editing aimed at promoting polyketide synthesis (Yang et al., 2021). The CRISPR-Cas9 system was utilized to knock out the erythromycin biosynthetic gene cluster, resulting in the generation of a recombinant strain, Abery, which inhibits the synthesis of erythromycin while accumulating significant amounts of the synthetic substrates methylmalonate-CoA and malonate coenzyme A. Using Abery as a heterologous host, three genes—*AsCHS*, *RgTAL*, and *Sc4CL*—were introduced to facilitate the production of new polyketides from L-tyrosine and methylmalonate-CoA (Ren et al., 2021). And a review discusses the application of CRISPR/Cas9 technology in the synthesis of secondary metabolites, including polyketides, in filamentous fungi. Furthermore, CRISPR/Cas9 technology has been employed to enhance the production of polyketides, such as increasing the production of the plant growth hormone gibberellic acids through gene editing. The researchers also explored the potential of utilizing CRISPR/Cas9 technology to activate silent gene clusters within the polyketide biosynthetic pathway (Jiang et al., 2021). A customized pCRISPR–Cas9apre system was developed to enhance the biosynthesis of ansamitocin P-3 (AP-3), a polyketide, in Actinosynnema pretiosum. Through CRISPR–Cas9-mediated gene editing, the researchers deleted gene clusters that compete with the AP-3 biosynthetic pathway and enhanced the supply of “glycolate” elongation units by inserting bidirectional promoters (BDPs). These strategies successfully increased AP-3 production (Guo S. et al., 2022). Additionally, the researchers established an efficient CRISPR/Cas9-mediated gene knockout system for Trichoderma hamatum T21, a filamentous fungus. They effectively knocked out the polyketide synthase gene (PKS) associated with pigment synthesis, facilitating the investigation of the fungus’s biological control mechanisms and the synthesis of secondary metabolites. The article discusses methods for editing the type I polyketide synthase (PKS) gene *in vitro* using the CRISPR-Cas9 system. The researchers successfully heterologously expressed the edited biosynthetic gene cluster in *E. coli*, resulting in the production of the desired polyketide derivatives. This technology provides a platform for the rational design of natural product derivatives for future drug development (Luo et al., 2023; Kudo et al., 2020). Furthermore, an article reviews the application of the CRISPR/Cas system in Streptomyces, with a particular emphasis on the identification and activation of silent polyketide biosynthetic gene clusters (smBGCs) through CRISPR/Cas technology. It summarizes the application of the CRISPR/Cas system in Streptomyces and discusses its potential for natural product discovery and production (Lee et al., 2024).

The application of CRISPR technology in plant secondary metabolites offers innovative solutions for the sustainable development of agriculture. By enhancing crop disease resistance, optimizing nutritional composition, and improving stress tolerance, CRISPR technology not only increases agricultural production efficiency but also contributes significantly to environmental protection and ecological balance. Looking ahead, as technology continues to advance, CRISPR is poised to play an increasingly vital role in the sustainable development of global agriculture.

## 4 Prospects and future challenges of CRISPR in the synthesis of secondary metabolites

**Figure 2:**
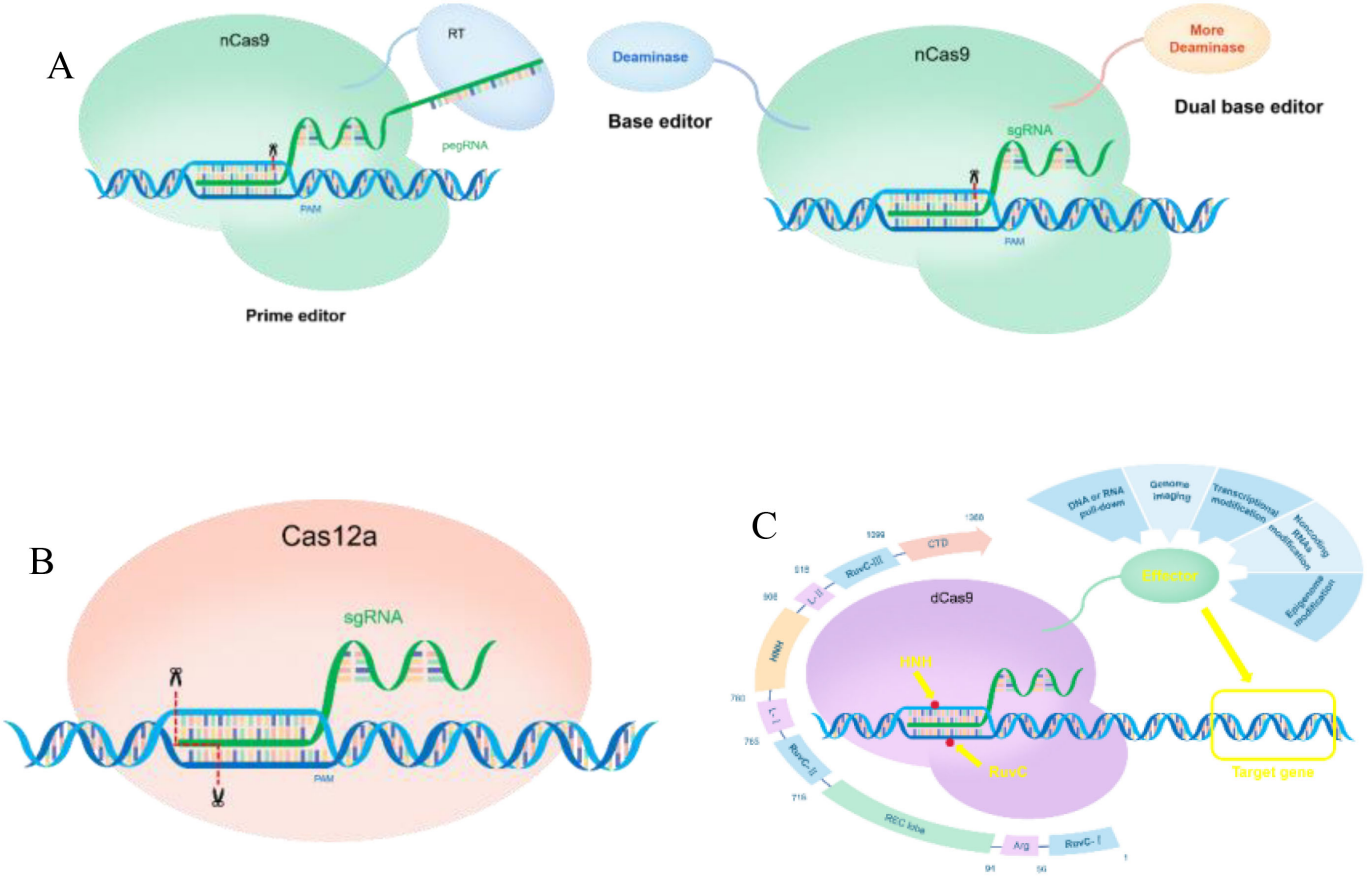
Application of CRISPR-a and CRISPRi in medicinal plants **(A)** The mechanism of action of the nCas9 base editor and gene editing. **(B)** Mechanism of action of Cas12a in gene regulation. **(C)** Application of dCas9 in the regulation of gene expression in plants.

Increasing the levels of active ingredients in medicinal plants is a primary objective in secondary plant metabolism research, as it serves as a critical indicator of their medicinal value. Achieving this objective necessitates the implementation of efficient, direct, and rapid gene editing technologies. Among the various gene editing methods, CRISPR stands out as a powerful tool that plays a vital role in precisely targeting the genomes of medicinal plants and facilitating the discovery and elucidation of the complex metabolic networks involved in the production of active compounds. To tackle practical research challenges, highly specific sgRNA design tools and various suitable mutant Cas proteins have been developed, including nCas9 for constructing base editors. The nCas9 protein can be utilized for larger fragment substitutions, significantly enhancing the mutation rate and effectively addressing the issue of low efficiency associated with homology-directed repair (HDR) in induced plants (**Figure 2A**; Ran et al., 2013). As a method of CRISPR interference (CRISPRi), dCas9 technology can inhibit gene transcription without altering the coding DNA sequence. This efficient gene expression regulation tool not only effectively reduces the expression of target genes but also provides a robust means to investigating the function of gene promoters and their roles within the intricate gene regulatory network (**Figure 2C**; Larson et al., 2013). Researchers have systematically investigated genome cutting activity and cytosine base editing efficiency in rice using codon-optimized variants such as ScCas9, ScCas9+, and ScCas9++, as well as the ScCas9n++ nickase variant. Furthermore, the researchers have developed a novel cytosine base editor, PevoCDA1-ScCas9n++, which is capable of achieving stable and efficient multiplex site-specific base editing at NNG-PAM sites. Additionally, they have developed a novel dual-base editor toolkit called PhieDBEs, which includes highly efficient dual-base editors containing single-chain DNA-binding domains (DBDs) and offers a broad targeting range suitable for plants. By analyzing the performance of these editors at 48 targets in rice, the researchers found that the DBE-SpGn construct, containing a single DBD and a deaminase located at the N-terminus of SpGn, also exhibited the highest editing efficiency (Zheng et al., 2024; Liu et al., 2021; Tan et al., 2023). In addition to the basic CRISPR-Cas9 technology, several modified and enhanced systems have been developed. For instance, a research group introduced the Cpf1 system, in 2015 as an alternative genome-editing tool within the CRISPR-Cas family (Zetsche et al., 2015). The Cas12a-crRNA complex serves as an efficient genome-editing tool for plant genomics, requiring only the automatic processing of crRNA and the formation of sticky ends upon cleavage. This characteristic facilitates the insertion and deletion of large fragments more easily than the blunt ends produced by Cas9 (**Figure 2B**; Jiang, 2019, 4-5; Zhang et al., 2024). CRISPR-Cas12a has been utilized for enzyme inhibition to manipulate plant metabolites. Researchers have demonstrated the effective enzymatic chemistry of the CRISPR-Cpf1 system in soybean and tobacco, inducing mutations in the *FAD2-1B* and *FAD2-1A* genes, which promotes the conversion of oleic acid to linoleic acid and enhances the nutritional value of soybean (Kim et al., 2017). Collectively, these advancements have established a robust foundation for the application of CRISPR systems in plant research.

Current research on non-coding RNA regulation in medicinal plants primarily focuses on the editing of miRNA and lncRNA. miRNAs predominantly negatively regulate gene expression at both the transcriptional and post-transcriptional levels, while lncRNAs are involved in various growth and developmental processes in plants (Li et al., 2022; Zetsche et al., 2020). In recent years, the regulation of non-coding RNA has been applied to nearly 30 Chinese medicinal plants, including Artemisia annua, safflower, ddendrobium, ginseng, Panax notoginseng, and Hulanhuang, to investigate their significant metabolic activities (Li et al., 2011; Meng et al., 2016; Pani et al., 2011; Vashisht et al., 2015; Wei et al., 2015; Wu et al., 2012). The editing of miRNA typically involves disrupting the transcriptional start site or other functional binding sites, or applying mutations to the miRNA binding sites in target genes to confirm targeting. In contrast, the editing of lncRNA often focuses on altering the length of the gRNA used for guidance. Notably, as early as 2015, CRISPR technology was successfully employed to targe and edited non-coding RNA in soybeans, utilizing a gene gun to deliver sgRNA/Cas9 vectors to target genes (Jacobs et al., 2015). This technology has also been employed to knock out genes associated with tomato ripening, thereby inhibiting the ripening process in mutant strains of tomatoes (Li et al., 2018). As CRISPR technology continues to evolve and innovates, we anticipate the emergence of more sophisticated non-coding RNA editing technologies, highlighting its promising applications in medicinal plants.

Epistatic modification plays a crucial role in gene expression programs, with DNA methylation serving as one of the key mechanisms. The level of DNA methylation significantly influences various aspects of plant growth and development, as well as metabolic processes and reproduction. This phenomenon has been studied in several medicinal plants, including willow puncturefis, dendrobium, woad, ginseng, and salvia (Cubas et al., 1999; Li et al., 2015; Ni et al., 2014; Yang et al., 2013; Zhang et al., 2012). In conclusion, utilizing DNA methylation to regulate the secondary metabolism of medicinal plants represents an effective strategy.

However, compared to other research areas in molecular biology, the molecular research on medicinal plants is limited, primarily due to three reasons. First, the sequencing of medicinal plant genomes is not yet mature, and the application of CRISPR technology is influenced by various factors, with the sequencing of the genome and the integrity of the data being the primary factors affecting experimental validity. Nevertheless, most medicinal plants with sequenced genomes and explored secondary metabolism using CRISPR technology are those with simple genetic transformation systems, while research on medicinal plants with complex genomes and difficult genetic transformation is still weak. This makes the application of CRISPR technology in medicinal plants with unknown genomes speculative. Second, there is a lack of genetic transformation systems for medicinal plants. In the absence of genomic data, establishing a genetic transformation system for medicinal plants is challenging, and the accuracy of the established systems is less than ideal. Currently, only a fraction of medicinal plants, such as *Salvia miltiorrhiza* and Panax ginseng, have well-established genetic transformation systems, and the ability of transformed plant cells to regenerate into whole plants is a significant challenge. Many plants are difficult to regenerate from a single cell or tissue, limiting the ultimate application of gene editing. Third, while CRISPR technology is a powerful gene-editing tool, there are still many technical issues that need to be improved, such as off-target effects, editing efficiency, and the restrictiveness of PAM sequences. These are the main directions for the future development and optimization of CRISPR technology.

As the CRISPR toolbox continues to expand, a diverse array of strategies will be employed to investigate plant secondary metabolic pathways in the future. We summarize three prospective aspects as follows:

### 4.1 CRISPR-based regulation technologies for plant secondary metabolic pathway optimization

The secondary metabolism network of active substances in medicinal plants often necessitates interactions between different pathways, leading to the production of medicinal active substances being influenced by the expression levels of multiple genes within these pathways. To further analyze the impact of gene networks on secondary metabolites or to enhance yields more precisely, it is essential to fine-tune the activities of relevant intermediate enzymes, ensuring that each reaction is compatible with the overall biosynthetic pathway. Currently, CRISPR-based strategies for secondary metabolite research typically involve knocking out or significantly altering the expression of target genes, rather than enabling precise regulation. However, researchers are now capable of fine-tuning gene activity in model plants. For instance, by inducing various mutations in non-coding regions, such as uORF regions, 5’UTR, and 3’UTR, a range of product levels can be generated, resulting in plants with the desired product content (Huang et al., 2020; Wang et al., 2024; Xing et al., 2020; Xue et al., 2023; Zeng et al., 2020). Similarly, introducing a series of mutations in amino acids can lead to the fine-tuning of enzyme activity (Xu et al., 2021). These methods have been validated and applied in model plants and may also serve as important strategies for enhancing medicinal plants.

In comparison to non-coding sequence editing, CRISPRa/i and epigenetic editing facilitated by the CRISPR system offer a straightforward, direct, and flexible approach, particularly for the simultaneous regulation of multiple genes. Enhancing the precision of CRISPRa/i regulation levels and CRISPR-based epigenetic regulation—such as the modification of regulatory elements or the engineering of guide RNA structures—can facilitate the quantitative expression of target genes to manageable levels. Moreover, current strategies for regulating secondary metabolic networks typically involve the editing or modulation of a single or a limited number of intermediate enzymes, rather than a comprehensive series of genes within the entire network. With a deeper understanding of each biological process and the rapid advancements in multiplex gene editing technology, varying degrees of simultaneous gene regulation will address challenges in plant pharmaceutical engineering, enabling production levels that surpass those achievable through traditional methods.

### 4.2 CRISPR combined with high-throughput technology enable rapid understanding of secondary metabolite synthesis networks

Currently, our understanding of the gene networks responsible for the production of most active ingredients in botanicals remains limited, as the functions of many genes are still unknown. CRISPR-based high-throughput screening is poised to significantly enhance the discovery of gene functions through reverse genetic screening. The establishment of CRISPR knockout (CRISPR-KO), CRISPR activation (CRISPRa), and CRISPR interference (CRISPRi) libraries enables high-throughput knockout, activation, and knockdown of genes, thereby influencing product yields. By leveraging the consistent one-to-one correspondence between product yields and the sgRNA used in CRISPR, critical unknown genes can be rapidly identified. However, the successful application of CRISPR-based high-throughput screening is contingent upon the development of efficient genetic transformation systems. In the future, advancements in genetic transformation technology and plant cell culture methods are expected to enhance CRISPR-based high-throughput screening strategies, providing significant support for individual-level gene discovery, analysis, regulation, and the utilization of plant cell factories. This progress will greatly facilitate the optimization and modification of secondary metabolic pathways, as well as improve the capability to transfer gene pathways across different species (Liu et al., 2023; Shalem et al., 2015).

### 4.3 CRISPR-based large DNA fragment manipulation for biological chassis optimization and plant molecular farming application

Currently, CRISPR technology has enabled the editing of large DNA fragments and chromosomes, offering a novel strategy for investigating the functions of genes associated with secondary metabolites. For instance, CRISPR-based tools for large deletions and replacements facilitate the deletion of gene clusters, as well as the exchange of promoters and genes. Furthermore, CRISPR can induce chromosomal rearrangements, including interchromosomal rearrangements, inversions, deletions, and truncations, allowing for the rapid simplification and modification of plant biological chassis. This capability can be employed to eliminate toxic by-products or redundant sequences, remove negative regulators that hinder cell or plant growth, or upregulate endogenous plant processes that enhance the production of secondary metabolites. Additionally, it accelerates the construction of minimal genomes or modifies them to be more suitable as biofactories. Building on the optimization of the chassis, CRISPR-based targeted large DNA sequence integration tools, such as the newly developed Prime Editing technology, can be utilized for the swift transfer of exogenous secondary metabolism-related elements or entire pathways to address deficiencies or key components in the chassis gene. This advancement facilitates the stable and efficient production of target metabolites in plant molecular agriculture (Sun et al., 2024; Zhu et al., 2022). Moreover, CRISPR-based DNA fragment manipulation technology allows for the straightforward testing and optimization of pathways, further enhancing the yield of molecular agriculture products. Consequently, the development of robust CRISPR-based systems for operating on large DNA fragments and their applications will be a significant aspect of CRISPR’s role in the biosynthesis of bioactive compounds from plants in the future.

## 5 Conclusion

Previous studies have conducted a comprehensive review of medicinal and aromatic plants, outlining the advantages and disadvantages of CRISPR technology in comparison to other methods. These studies further examines the application of CRISPR technology in the functional studies of medicinal plant genomes, genetic enhancement, synthetic biology, and germplasm innovation. Additionally, research investigates the role of these technologies in enhancing crop nutritional value, productivity, and resilience to biotic and abiotic stresses (Jamwal et al., 2023; Saha et al., 2024; Das et al., 2024).

This article focuses on the application and development of CRISPR technology concerning secondary metabolites in medicinal plants, summarizes the regulatory strategies, and anticipates future research directions in this area. Additionally, it examines the potential applications of novel CRISPR technology in medicinal contexts. In conclusion, the ongoing optimization of the CRISPR system is enhancing the maturity of this technology, which presents broad application prospects. It offers essential technical support for the exploration of medicinal plants and has the potential to bring about significant changes in life sciences and technology. As CRISPR technology continues to advance, future research may concentrate on several key areas: Studies could utilize CRISPR technology to target multiple genes simultaneously, facilitating comprehensive regulation of complex metabolic pathways to improve the synthesis efficiency and diversity of secondary metabolites. The integration of genomic selection methods with CRISPR technology could enable more precise selection of desirable traits during the breeding process, thereby accelerating the genetic enhancement of plants. Furthermore, advancements in CRISPR technology may allow for the transfer and integration of genes associated with the synthesis of secondary metabolites across different species, thereby enabling the production of desired secondary metabolites in a wider variety of plant species.
